# Prognostic factors and survival in elderly breast cancer patients: roles of age, stage and inflammatory markers

**DOI:** 10.1186/s12885-026-16142-8

**Published:** 2026-05-07

**Authors:** Doğancan Akpalamut, Halil İbrahim Ellez, Elif Atağ, Oktay Halit Aktepe, Hüseyin Salih Semiz, Aziz Karaoğlu

**Affiliations:** 1 Department of Internal Medicine, Dokuz Eylul University, İzmir, 35390 Turkey; 2 Şanlıurfa Mehmet Akif İnan Research and Training Hospital, Şanlıurfa, 63050 Turkey; 3 Institute of Oncology, Dokuz Eylul University, Izmir, 35340 Turkey; 4 Department of Medical Oncology, Faculty of Medicine, Dokuz Eylul University, İzmir, 35340 Turkey

**Keywords:** Elderly breast cancer, Geriatric oncology, ECOG performance status, Charlson Comorbidity Index, DNLR, Prognostic factors

## Abstract

**Background:**

Breast cancer is a major clinical problem in elderly patients, yet data on prognostic factors and treatment outcomes in this population remain limited. This study investigated clinical characteristics, treatment patterns, and survival in elderly breast cancer patients, with focus on functional status, comorbidity burden, and inflammatory markers.

**Methods:**

This retrospective study included 261 patients aged ≥ 65 years diagnosed with breast cancer at Dokuz Eylül University Medical Oncology Clinic between 2010 and 2023. Patients were stratified into two age groups (65–74 and ≥ 75 years). Clinicopathological characteristics, treatment modalities, ECOG performance status, Charlson Comorbidity Index (CCI), and the derived neutrophil-to-lymphocyte ratio (dNLR) were analyzed. Treatment comparisons were restricted to non-metastatic disease (stage I–III, *n* = 204). Survival was analyzed with Kaplan–Meier and log-rank tests. Receiver operating characteristic (ROC) analysis identified an optimal dNLR cutoff, and time-dependent ROC with inverse probability of censoring weighting assessed discriminatory performance over time. Multivariate Cox regression with backward likelihood ratio elimination identified independent prognostic factors; Harrell’s concordance index was calculated for model validation.

**Results:**

Median follow-up was 142 months. No significant differences were observed between age groups regarding pathological features, receptor status, or comorbidities. However, patients aged ≥ 75 years were less likely to undergo primary surgery (*p* = 0.041), adjuvant chemotherapy (*p* = 0.001), or radiotherapy (*p* = 0.001). Median overall survival was 148 versus 87 months (*p* < 0.001). ROC identified an optimal dNLR cutoff of 1.74 (AUC 0.628); time-dependent AUCs were 0.772, 0.652, and 0.668 at 1, 3, and 5 years. LDH showed no significant discriminatory capacity. Multivariate analysis identified ECOG ≥ 2 (HR 2.89), CCI ≥ 3 (HR 2.12), stage IV (HR 7.43), dNLR > 1.74 (HR 2.14), TNBC (HR 2.30), age ≥ 75 (HR 1.52), and surgery (HR 0.53) as independent predictors of mortality. The full model achieved a C-index of 0.792.

**Conclusion:**

Elderly breast cancer patients are less frequently treated despite similar clinicopathological features. ECOG performance status, CCI, disease stage, dNLR, and clinical subtype are independent predictors of mortality. The prognostic impact of chronological age was substantially attenuated after adjusting for functional status and comorbidity, emphasizing that treatment decisions should be guided by comprehensive geriatric assessment rather than age alone.

Article.

## Introduction

Breast cancer is the most commonly diagnosed cancer and the leading cause of cancer death among women worldwide [1]. Incidence continues to rise, particularly among older adults, with the highest rates observed in women aged 70–79 years [2]. Moreover, the percentage of those who are diagnosed with breast cancer in the elderly age group is expected to increase. Limited data are available for those patients because clinical trial data that predominantly involve younger patients do not provide sufficient information to accurately assess the outcomes of therapy in older adults [3].

Early diagnosis of breast cancer and timely, effective implementation of treatment strategies are crucial for reducing breast cancer-related mortality. Several factors, including patient age, life expectancy, comorbidities, disease stage and molecular markers, play a significant role in determining the most appropriate treatment approach [4]. In addition to hormone status, stage and age, inflammatory markers such as the dNLR (derived neutrophil-to-lymphocyte ratio) have prognostic value in these patients. In one study, high dNLRs and LDH levels were related to worse outcomes in HER2-positive breast cancer patients [5]. Moreover, the dNLR can reflect the immune state of a disease, and its prognostic importance has been shown in several studies [6, 7]. Other inflammatory markers, such as lactate dehydrogenase (LDH), can have prognostic importance and can be used to evaluate treatment efficacy [8, 9]. Despite these insights, factors influencing overall survival in elderly patients with breast cancer remain poorly defined, particularly regarding the role of serum biomarkers in guiding treatment decisions and surveillance strategies. This study aimed to investigate the clinical characteristics and determinants of overall survival in elderly patients with breast cancer. Given that comorbid conditions in this population may substantially impact survival, we also sought to examine nonmalignant factors that contribute to mortality.

## Methods

### Patient selection

This retrospective study included patients aged 65 years or older who were diagnosed with breast cancer at Dokuz Eylül University Medical Oncology Clinic between 2010 and 2023. Patients diagnosed before the age of 65 and those with no follow-up data beyond the initial diagnosis (*n* = 9) were excluded, leaving 261 patients for analysis. Patients who had at least one clinical follow-up visit but were subsequently lost to follow-up were censored at the date of their last recorded visit and retained in all survival analyses. Demographic, clinical, and pathological characteristics as well as treatment details were collected in accordance with the ethical approval obtained from the Dokuz Eylül University Ethics Committee.

### Clinical characteristics and data collection

Patients’ ages and stages at the time of diagnosis, hormonal status, and grades were collected. Data on surgical procedures, neoadjuvant and adjuvant chemotherapies, hormonal therapies, and anti-HER2 treatments were documented. Recurrence, metastatic status, and first-, second-, and third-line treatment regimens were also recorded. The primary endpoint was overall survival (OS), defined as the time from diagnosis to death from any cause or censoring at last follow-up. Patients were stratified into two different age groups: Group 1 (65–74 years), Group 2 (≥ 75 years). The Charlson Comorbidity Index (CCI) was calculated for each patient based on available comorbidity data, including diabetes mellitus, coronary artery disease, chronic pulmonary disease, chronic kidney disease, dementia, and musculoskeletal disease. Patients were categorized into three CCI groups: 0, 1–2, and ≥ 3. Moreover, treatment comparisons regarding surgical intervention and adjuvant therapies were restricted to patients with non-metastatic disease (stage I–III, *n* = 204), excluding those with stage IV disease (*n* = 40) and unknown stage (*n* = 17).

Eastern Cooperative Oncology Group (ECOG) performance status at the time of diagnosis was retrospectively assessed from clinical records. ECOG scores were available for 232 patients (88.9%).

### Blood parameters

LDH levels and complete blood counts were recorded before treatment initiation. The derived neutrophil-to-lymphocyte ratio (dNLR) was calculated as neutrophil/(leukocytes – neutrophils). The NLI score was defined as an elevated LDH (above the upper normal limit) combined with the dNLR, classifying patients into three risk groups (0: low, 1: intermediate, 2: high).

### Statistical analysis

Descriptive statistics were calculated using SPSS version 29.0. Continuous variables are reported as medians (range) or means (standard deviation), and categorical variables as frequencies (percentages). Differences between age groups were compared using the chi-square or Fisher’s exact test. Overall survival was estimated using the Kaplan–Meier method, and differences between groups were compared with the log-rank test (R version 4.3.1; R Foundation for Statistical Computing, Vienna, Austria). Optimal cutoff values for dNLR and LDH were determined by receiver operating characteristic (ROC) curve analysis. Time-dependent ROC curves were estimated with inverse probability of censoring weighting using the timeROC R package (version 0.4) to assess discrimination at 1, 3, and 5 years. Independent prognostic factors were identified by multivariate Cox proportional hazards regression with backward likelihood ratio elimination. All covariates were entered as categorical variables: age group (65–74 vs. ≥75), ECOG (0–1 vs. ≥2), CCI (0, 1–2, ≥ 3), dNLR (≤ 1.74 vs. >1.74 at the ROC-derived cutoff), stage (I–IV, with Unknown retained as a separate category), clinical subtype (HR+/HER2−, HER2+/HR−, HR+/HER2+, TNBC), surgical status (yes/no), adjuvant chemotherapy (yes/no), and adjuvant radiotherapy (yes/no). The most favorable prognostic group was used as the reference for each variable. Model discrimination was evaluated with Harrell’s concordance index (C-index) using the survival R package (version 3.5–7.5). A two-sided *p* value < 0.05 was considered statistically significant for all analyses.

## Results

### Characteristics of the age groups

A total of 261 patients were included in this study, and the median follow-up was 142 months (95% CI 130.3–153.3). Patients were stratified into two age groups: 65–74 years (*n* = 145) and ≥ 75 years (*n* = 116). Among patients aged 65–74 years, 4 (2.8%) were male, whereas 2 (1.7%) of those aged ≥ 75 years were male.

The clinical and pathological characteristics of the age groups are summarized in Table 1.

**Table 1:** Pathological and clinical characteristics of the age groups

Variable	Category	65–74 (*n* = 145)	≥ 75 (*n* = 116)	*p* value
ECOG (*n* = 232)	0–1	107 (83.6)	79 (76.0)	0.199
	≥ 2	21 (16.4)	25 (24.0)	
Diabetes Mellitus	Yes	52 (35.9)	32 (27.6)	0.197
Hypertension	Yes	85 (58.6)	82 (70.7)	0.059
Coronary Artery Disease	Yes	48 (33.1)	40 (34.5)	0.918
Chronic Pulmonary Disease	Yes	25 (17.2)	19 (16.4)	0.985
Chronic Kidney Disease	Yes	20 (13.8)	19 (16.4)	0.684
Cognitive Dysfunction	Yes	11 (7.6)	17 (14.7)	0.103
Musculoskeletal Disease	Yes	56 (38.6)	58 (50.0)	0.086
CCI	0	38 (26.2)	25 (21.6)	0.679
	1–2	71 (49.0)	61 (52.6)	
	≥ 3	36 (24.8)	30 (25.9)	
Stage	I	31 (21.4)	18 (15.5)	0.267
	II	46 (31.7)	30 (25.9)	
	III	43 (29.7)	36 (31.0)	
	IV	18 (12.4)	22 (19.0)	
	Unknown	7 (4.8)	10 (8.6)	
Histology	IDC	63 (43.4)	54 (46.6)	0.360
	ILC	28 (19.3)	28 (24.1)	
	Other	54 (37.2)	34 (29.3)	
Grade	1	13 (9.0)	14 (12.1)	0.347
	2	65 (44.8)	51 (44.0)	
	3	48 (33.1)	43 (37.1)	
	Unknown	19 (13.1)	8 (6.9)	
ER Status	Positive	118 (81.4)	97 (83.6)	0.758
	Negative	27 (18.6)	19 (16.4)	
PR Status	Positive	98 (67.6)	84 (72.4)	0.479
	Negative	47 (32.4)	32 (27.6)	
HER2 Status	Positive	23 (15.9)	11 (9.5)	0.181
	Negative	122 (84.1)	105 (90.5)	
Clinical Subtype	HR+/HER2-	107 (73.8)	91 (78.4)	0.208
	HER2+/HR-	12 (8.3)	4 (3.4)	
	TNBC	15 (10.3)	14 (12.1)	
	HR+/HER2+	11 (7.6)	7 (6.0)	
dNLR (*n* = 258)	≤ 1.74	90 (62.9)	63 (54.8)	0.231
	> 1.74	53 (37.1)	52 (45.2)	

*CCI* Charlson Comorbidity Index, *dNLR* derived neutrophil–lymphocyte ratio, *ECOG* Eastern Cooperative Oncology Group, *ER* estrogen receptor, *HER2* human epidermal growth factor receptor 2, *IDC* invasive ductal carcinoma, *ILC* invasive lobular carcinoma, *PR* progesterone receptor, *TNBC* triple-negative breast cancer

*P* values were calculated using chi-square or Fisher’s exact test

ECOG performance status was available for 232 patients (88.9%). The majority of patients in both age groups had an ECOG score of 0–1 (83.6% vs. 76.0%, *p* = 0.199). The median CCI score was 1 (range, 0–7), with no significant difference between the two age groups (*p* = 0.679). No statistically significant differences were observed between the age groups regarding pathological features, receptor status, clinical subtypes, or comorbidities. However, patients aged ≥ 75 years tended to be diagnosed at more advanced stages, although this did not reach statistical significance (*p* = 0.267).

Treatment comparisons were restricted to patients with non-metastatic disease (stage I–III, *n* = 204), excluding those with stage IV disease (*n* = 40) and unknown stage (*n* = 17). Among patients with stage I–III disease, surgical intervention was performed in 99.2% of those aged 65–74 years compared with 92.9% of those aged ≥ 75 years (*p* = 0.041). The administration of adjuvant chemotherapy (53.3% vs. 29.8%, *p* = 0.001) and adjuvant radiotherapy (91.7% vs. 72.6%, *p* = 0.001) also declined significantly in the older age group. No significant differences were observed regarding adjuvant hormonotherapy (*p* = 0.695) or neoadjuvant therapy (*p* = 0.420). These comparisons are summarized in Table 2.

**Table 2:** Treatment modalities in patients with stage I–III disease

Variable	Category	65–74 (*n* = 120)	≥ 75 (*n* = 84)	*p* value
Surgery	Yes	119 (99.2)	78 (92.9)	0.041
	No	1 (0.8)	6 (7.1)	
Adjuvant Chemotherapy	Yes	64 (53.3)	25 (29.8)	0.001
	No	56 (46.7)	59 (70.2)	
Adjuvant Radiotherapy	Yes	110 (91.7)	61 (72.6)	0.001
	No	10 (8.3)	23 (27.4)	
Adjuvant Hormonotherapy	No	25 (20.8)	15 (17.9)	0.695
	Tamoxifen	5 (4.2)	5 (6.0)	
	AI	77 (64.2)	58 (69.0)	
	AI+Tamoxifen	13 (10.8)	6 (7.1)	
Neoadjuvant Therapy	Yes	22 (18.3)	11 (13.1)	0.420
	No	98 (81.7)	73 (86.9)	

Treatment comparisons were restricted to patients with stage I–III disease (*n* = 204)

*AI* aromatase inhibitor

*P* values were calculated using chi-square or Fisher’s exact test

### ROC analysis

ROC curve analysis was performed to identify optimal cutoff values of dNLR and LDH for mortality prediction. The dNLR demonstrated statistically significant discriminatory capacity (AUC 0.628, 95% CI 0.560–0.696; *p* < 0.05), with an optimal cutoff of 1.74 (sensitivity 53.4%, specificity 74.8%). In contrast, LDH did not reach statistical significance (AUC 0.539, 95% CI 0.468–0.610; *p* = 0.212) and was therefore excluded from subsequent survival analyses (Table 3).

**Table 3:** ROC analysis of inflammatory markers for mortality analysis

Variable	AUC (95% CI)	Cutoff Value	Sensitivity (%)	Specificity (%)	*p* value
dNLR	0.628 (0.560–0.696)	1.74	53.4	74.8	< 0.05
LDH	0.539 (0.468–0.610)	243	21.0	93.9	0.212

*dNLR* derived neutrophil-to-lymphocyte ratio, *LDH* lactate dehydrogenase, *AUC* area under the curve

**Fig. 1:**
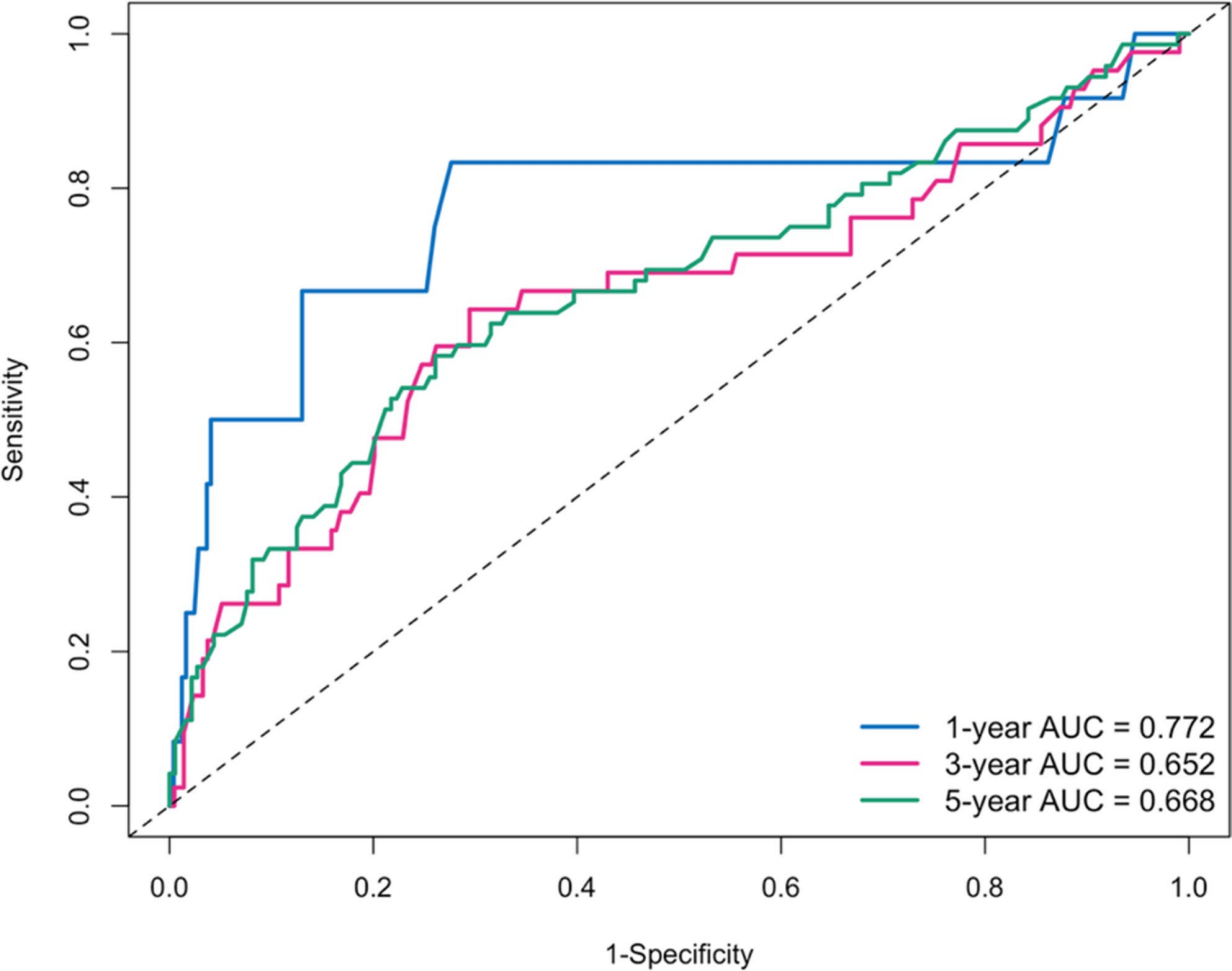
Time-dependent ROC curves for dNLR. Discriminatory performance of dNLR for mortality at 1, 3, and 5 years, estimated using inverse probability of censoring weighting. dNLR, derived neutrophil-to-lymphocyte ratio; AUC, area under the curve; ROC, receiver operating characteristic

To further evaluate the time-varying prognostic performance of dNLR, time-dependent ROC analysis was performed using inverse probability of censoring weighting. The dNLR demonstrated robust discrimination for mortality at 1 year (AUC 0.772, 95% CI 0.592–0.953), with sustained prognostic value at 3 years (AUC 0.652, 95% CI 0.550–0.754) and 5 years (AUC 0.668, 95% CI 0.590–0.747) (Fig. 1). Harrell’s concordance index for dNLR alone was C-index 0.620 (95% CI 0.578–0.662), while the full multivariate Cox model incorporating age group, stage, ECOG, CCI, dNLR, clinical subtype, and surgery achieved a C-index of 0.792 (95% CI 0.754–0.829), indicating strong overall discriminatory capacity. 

### Overall survival

**Fig. 2:**
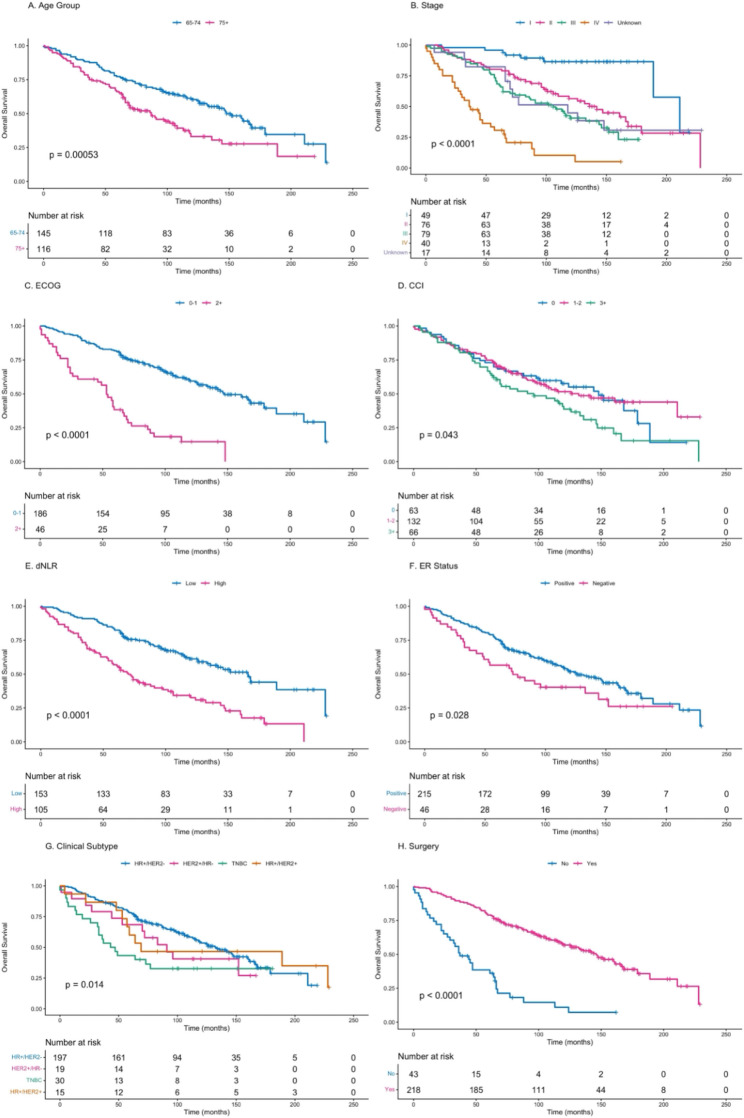
Kaplan–Meier analysis of overall survival. Survival curves stratified by age group (**a**), stage (**b**), ECOG performance status (**c**), CCI (**d**), dNLR (**e**), ER status (**f**), clinical subtype (**g**), and surgery (**h**) (p values by log-rank test)

In the survival analysis stratified by age group, the median overall survival was 148 months (95% CI, 126–211) in patients aged 65–74 years and 87 months (95% CI, 66–113) in those aged ≥ 75 years (*p* < 0.001) (Fig. 2A). A significant trend toward shorter survival was observed with increasing disease stage: 211 months (95% CI, 189–NR) for stage I, 142 months (95% CI, 106–179) for stage II, 106 months (95% CI, 74–144) for stage III, and 36.5 months (95% CI, 27–57) for stage IV (*p* < 0.001) (Fig. 2B).

Patients with an ECOG performance status of ≥ 2 had significantly worse OS compared with those with ECOG 0–1 (median 53 months (95% CI, 30–69) vs. 147 months (95% CI, 126–211); *p* < 0.001) (Fig. 2C). Higher comorbidity burden was also associated with poorer survival; patients with a CCI ≥ 3 had a median OS of 96 months (95% CI, 66–133), compared with 148 months for CCI 0 and 132 months for CCI 1–2 (*p* = 0.043) (Fig. 2D).

**Fig. 3:**
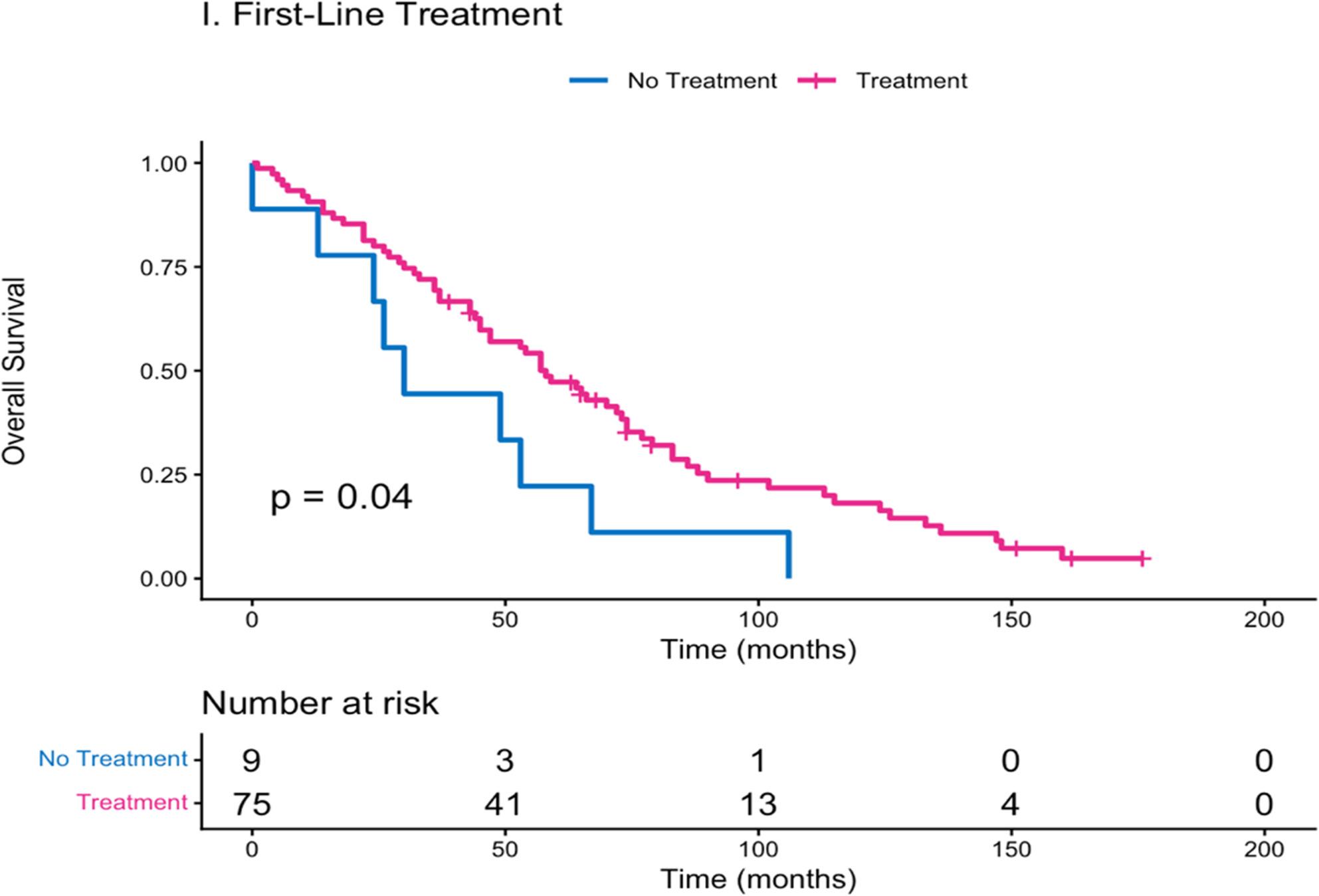
Kaplan–Meier analysis of overall survival by first-line treatment status in metastatic patients. Survival curves of patients with metastatic or recurrent disease (*n* = 84) stratified by receipt of first-line systemic treatment. *P* value by log-rank test

The dNLR was significantly associated with survival; median OS was 166 months (95% CI, 133–NR) for patients with a dNLR ≤ 1.74 and 70 months (95% CI, 57–98) for those with a dNLR > 1.74 (*p* < 0.001) (Fig. 2E). ER-positive patients demonstrated a longer median OS of 126 months (95% CI, 110–161) than ER-negative patients (73 months, 95% CI, 49–152; *p* = 0.028) (Fig. 2F). Overall survival varied significantly across clinical subtypes (*p* = 0.014), with HR+/HER2 − patients exhibiting the longest median OS (132 months), followed by HER2+/HR− (91 months), HR+/HER2+ (69 months), and TNBC (45 months) (Fig. 2G). Surgical intervention was also associated with significantly longer survival (146 vs. 37 months, *p* < 0.001) (Fig. 2H). No significant difference was observed based on PR status (PR-positive: 126 months (95% CI, 108–152) vs. PR-negative: 96 months (95% CI, 77–NR); *p* = 0.40). Grade was also not significantly associated with overall survival (*p* = 0.347), thus excluding multivariate analysis. Eighty-four patients had de novo metastatic disease or experienced recurrence/metastasis, 75 of whom (89.3%) received first-line treatment. Among those who received first-line treatment, 51 patients (68%) also received second- and third-line treatment. Patients who received first-line treatment had longer survival than those who did not (58 months (95% CI, 45–74) vs. 30 months (95% CI, 24–NR); *p* = 0.04) (Fig. 3).

In univariate analysis, advanced age, disease stage, ECOG ≥ 2, CCI ≥ 3, higher dNLR, ER negativity, clinical subtype, and surgical status were associated with worse prognosis. Grade and PR status were not significant. Multivariate Cox regression with backward likelihood ratio elimination identified disease stage, ECOG performance status, CCI, dNLR, clinical subtype, surgical status, and age group as independent predictors of mortality. ECOG ≥ 2 (HR 2.89, 95% CI 1.90–4.41, *p* < 0.001) and CCI ≥ 3 (HR 2.12, 95% CI 1.25–3.57, *p* = 0.005) were among the strongest prognostic factors. Adjuvant chemotherapy and radiotherapy were eliminated during backward selection, while surgical intervention remained independently associated with improved survival (HR 0.53, 95% CI 0.28–0.98, *p* = 0.044) (Table 4). 

**Table 4:** Univariate and multivariate analyses of survival

Characteristics		Univariate		Multivariate	
		Median OS, m (95% CI)	p	HR (95% CI)	p
Age Group			0.0005		0.037
	65–74	148 (126–211)		Reference	
	≥ 75	87 (66–113)		1.52 (1.03–2.25)	
ECOG			< 0.001		< 0.001
	0–1	147 (126–211)		Reference	
	≥ 2	53 (30–69)		2.89 (1.90–4.41)	
CCI			0.04		0.316 0.005
	0	148 (101–NR)		Reference	
	1–2	132 (98–NR)		1.28 (0.79–2.09)	
	≥ 3	96 (66–133)		2.12 (1.25–3.57)	
Stage			< 0.001		
	I	211 (189–NR)		Reference	0.002 < 0.001 < 0.001 0.010
	II	142 (106–179)		3.49 (1.61–7.58)	
	III	106 (74–144)		4.26 (1.94–9.34)	
	IV	36.5 (27–57)		7.43 (2.82–19.56)	
	Unknown	118 (70–NR)		3.71 (1.37–10.09)	
Clinical Subtype			0.01		
	HR+/HER2–	132 (115–161)		Reference	0.524 0.001 0.409
	HER2+/HR–	91 (70–NR)		1.25 (0.63–2.48)	
	TNBC	45 (33–NR)		2.30 (1.38–3.84)	
	HR+/HER2+	69 (57–NR)		1.37 (0.65–2.88)	
dNLR			< 0.001		< 0.001
	≤ 1.74	166 (133–NR)		Reference	
	> 1.74	70 (57–98)		2.14 (1.44–3.17)	
Grade			0.2		
	1	179 (110–NR)		—	
	2	124 (91–NR)		—	
	3	108 (74–144)		—	
Surgery			< 0.001		0.044
	No	37 (27–65)		Reference	
	Yes	146 (126–168)		0.53 (0.28–0.98)	
Adjuvant CT			0.03		*
	No	106 (74–146)		—	
	Yes	147 (126–NR)		—	
Adjuvant RT			< 0.001		*
	No	61 (44–77)		—	
	Yes	147 (126–179)		—	

Multivariate analysis was performed using Cox proportional hazards regression with backward likelihood ratio elimination

*CCI* Charlson Comorbidity Index, *CI* confidence interval, *dNLR* derived neutrophil–lymphocyte ratio, *ECOG* Eastern Cooperative Oncology Group, *HER2* human epidermal growth factor receptor 2, *HR* hazard ratio, *OS* overall survival, *NR* not reached, *TNBC* triple-negative breast cancer

* Adjuvant chemotherapy and adjuvant radiotherapy were eliminated during backward selection. Concordance index = 0.792

## Discussion

In our study, advanced disease stage, older age at diagnosis, ECOG performance status, CCI ≥3, surgery, TNBC disease and higher dNLR were identified as independent risk factors for mortality. We also observed that with increasing age, fewer patients were offered curative treatment modalities, including chemotherapy and radiotherapy. There were no significant differences in stage comparison between the age groups.

Previous studies have reported that elderly patients tend to present at more advanced stages and receive less intensive treatment. Lodi et al. [10] demonstrated that patients aged ≥ 80 years had larger tumors and a higher rate of metastatic disease at presentation (8.0% vs. 5.9%, *p* < 0.01) compared with those aged 70–79 years. Consistent findings of reduced use of chemotherapy and radiotherapy in the oldest age groups have been reported in population-based studies [11, 12]. Although our cohort did not show a statistically significant stage difference between age groups, the observed undertreatment may reflect barriers to healthcare access, comorbidity burden, or fatalistic attitudes toward illness in later life.

No significant differences were observed between age groups regarding histological type, receptor status, or inflammatory markers. However, patients aged ≥ 75 years were less likely to undergo primary surgery (92.9% vs. 99.2%, *p* = 0.041), receive adjuvant chemotherapy (29.8% vs. 53.3%, *p* = 0.001), or adjuvant radiotherapy (72.6% vs. 91.7%, *p* = 0.001). Consistent with Bastiaannet et al. [12], who reported similar undertreatment in a population-based cohort of 127,805 patients, hormonotherapy was the preferred modality in our older subgroup (69% received aromatase inhibitors), likely reflecting its favorable tolerability profile. No significant difference in neoadjuvant therapy was observed between age groups. The lower rates of treatment administration among geriatric patients may be attributed to shorter life expectancy, comorbidity burden, concerns about treatment-related toxicity, and patient or physician preferences.

ROC analysis identified a dNLR cutoff of 1.74 with modest but statistically significant discriminatory capacity for mortality. The prognostic value of NLR in breast cancer is well-established; a systematic review by Corbeau et al. [13] confirmed dNLR as an independent mortality predictor, and both Geng et al. [14] and Krenn-Pilko et al. [15] reported an independent association between elevated NLR and poorer survival. In our multivariate analysis, elevated dNLR remained independently associated with worse OS, suggesting that inflammatory markers retain prognostic utility even in elderly patients, where age-related immune alterations might otherwise attenuate their relevance. Biologically, the prognostic value of dNLR reflects a pro-tumor inflammatory state in which neutrophils are recruited to the tumor microenvironment, where they physically interact with breast cancer cells to promote tumor proliferation, invasion, and angiogenesis [16]. Lymphopenia, conversely, reflects impaired anti-tumor immunity, and this imbalance may be further amplified in elderly patients by immunosenescence and inflammaging [17].

In contrast, LDH did not achieve significant discriminatory capacity in our cohort and was excluded from survival analysis, along with the NLI index, likely because these markers are not cancer-specific and can be influenced by numerous non-malignant factors. Although Li et al. [5] previously reported an optimal LDH cutoff of 244 U/L (AUC 0.793) in HER2-positive metastatic breast cancer patients treated with trastuzumab emtansine, our cohort included relatively few metastatic patients, which may have limited the prognostic signal of LDH. Further prospective studies are needed to clarify its role in this population.

ECOG performance status and CCI stood out as strong prognostic markers in our cohort. ECOG ≥ 2 was the single strongest predictor of mortality, which is in line with previous studies showing that functional status is one of the most important prognostic factors in elderly cancer patients [18]. CCI also showed independent prognostic value, consistent with earlier findings in elderly breast cancer [19]. After adjusting for ECOG and CCI, the effect of chronological age itself became much weaker. This suggests that in older patients, it is functional reserve and comorbidity burden that drive outcomes rather than age alone. Our findings support the SIOG and EUSOMA recommendations that treatment decisions in this population should be based on comprehensive geriatric assessment rather than age alone [20].

The strengths of this study include a relatively long median follow-up, single-center management ensuring consistent treatment approaches, and comprehensive evaluation of clinical, treatment, inflammatory, and survival parameters. Limitations include the retrospective single-center design, which may limit generalizability, and incomplete data on some CCI components (peripheral vascular disease, cerebrovascular disease, peptic ulcer) that were not systematically documented. ECOG was retrospectively assessed from clinical records and 11.1% of the patient records were missing. The study period (2010–2023) spans major therapeutic advances, including the introduction of CDK4/6 inhibitors and adjuvant abemaciclib, and only a minority of metastatic patients were treated in the post-CDK4/6 era, which may have attenuated subtype-specific survival differences. The sample size may be insufficient for detailed subgroup analyses, and competing-risk analysis distinguishing cancer-specific from non-cancer mortality was not performed. Future studies with larger multicenter cohorts could develop and externally validate a prognostic nomogram incorporating these factors to guide individualized treatment decisions in elderly breast cancer patients.

## Conclusions

In this cohort of elderly breast cancer patients, advancing age was associated with significant undertreatment despite comparable clinicopathological profiles. ECOG performance status and CCI emerged as the dominant prognostic determinants, substantially attenuating the independent effect of chronological age on mortality. These findings underscore that biological fitness should drive therapeutic decision-making, reinforcing the role of comprehensive geriatric assessment in optimizing outcomes for this growing patient population.

## Data Availability

The data used in this study are not publicly available because they contain confidential patient information. However, the datasets can be shared by the corresponding author upon reasonable request, provided that approval is obtained from the institutional ethics committee.

## Abbreviations

AI: Aromatase inhibitor
AUC: Area under the curve
CCI: Charlson Comorbidity Index
CI: Confidence interval
dNLR: Derived neutrophil-to-lymphocyte ratio
ECOG: Eastern Cooperative Oncology Group
ER: Estrogen receptor
EUSOMA: European Society of Breast Cancer Specialists
HER2: Human epidermal growth factor receptor 2
HR: Hazard ratio
IDC: Invasive ductal carcinoma
ILC: Invasive lobular carcinoma
LDH: Lactate dehydrogenase
NLI: Neutrophil-to-lymphocyte index
NLR: Neutrophil-to-lymphocyte ratio
NR: Not reached
OS: Overall survival
PR: Progesterone receptor
ROC: Receiver operating characteristic
SIOG: International Society of Geriatric Oncology
TNBC: Triple-negative breast cancer
